# Caspase-cleaved tau is senescence-associated and induces a toxic gain of function by putting a brake on axonal transport

**DOI:** 10.1038/s41380-022-01538-2

**Published:** 2022-04-07

**Authors:** Christian Conze, Marina Rierola, Nataliya I. Trushina, Michael Peters, Dennis Janning, Max Holzer, Jürgen J. Heinisch, Thomas Arendt, Lidia Bakota, Roland Brandt

**Affiliations:** 1 Department of Neurobiology, Osnabrück University, Osnabrück, Germany; 2 Center for Cellular Nanoanalytics, Osnabrück University, Osnabrück, Germany; 3 Center for Neuropathology and Brain Research, Paul Flechsig Institute of Brain Research, University of Leipzig, Leipzig, Germany; 4 Department of Genetics, Osnabrück University, Osnabrück, Germany; 5 Institute of Cognitive Science, Osnabrück University, Osnabrück, Germany

**Keywords:** Neuroscience, Cell biology

## Abstract

The microtubule-associated protein tau plays a central role in tauopathies such as Alzheimer’s disease (AD). The exact molecular mechanisms underlying tau toxicity are unclear, but aging is irrefutably the biggest risk factor. This raises the question of how cellular senescence affects the function of tau as a microtubule regulator. Here we report that the proportion of tau that is proteolytically cleaved at the caspase-3 site (TauC3) doubles in the hippocampus of senescent mice. TauC3 is also elevated in AD patients. Through quantitative live-cell imaging, we show that TauC3 has a drastically reduced dynamics of its microtubule interaction. Single-molecule tracking of tau confirmed that TauC3 has a longer residence time on axonal microtubules. The reduced dynamics of the TauC3-microtubule interaction correlated with a decreased transport of mitochondria, a reduced processivity of APP-vesicle transport and an induction of region-specific dendritic atrophy in CA1 neurons of the hippocampus. The microtubule-targeting drug Epothilone D normalized the interaction of TauC3 with microtubules and modulated the transport of APP-vesicles dependent on the presence of overexpressed human tau. The results indicate a novel toxic gain of function, in which a post-translational modification of tau changes the dynamics of the tau-microtubule interaction and thus leads to axonal transport defects and neuronal degeneration. The data also introduce microtubule-targeting drugs as pharmacological modifiers of the tau-microtubule interaction with the potential to restore the physiological interaction of pathologically altered tau with microtubules.

## Introduction

The microtubule-associated protein tau is an abundant neuronal protein that plays a central role in the pathology of a class of neurodegenerative diseases collectively called tauopathies [1]. This group of diseases includes Alzheimer’s disease (AD) as the most common one. Tauopathies are characterized by intracellular neurofibrillary tangles (NFTs) consisting of hyperphosphorylated tau protein and a disturbance of microtubule organization and microtubule-associated functions such as axonal transport. Remarkably, loss of tau does not lead to a major phenotypic change after chronic or acute knockout in cells or animals [2, 3]. This proves that—contrary to popular belief—tau’s primary role is not to stabilize microtubules in vivo. In turn this suggests that tau contributes to pathology by a (toxic) gain of function rather than a loss of function mechanism. This view is also supported by observations that tau reduction improves the symptoms of several neurological diseases [4–8]. It should be noted, however, that mice with a complete knockout of tau developed significant motor and cognitive deficits that are dependent on genetic background, suggesting that excessive lowering of tau should be avoided in therapeutic strategies for AD [9–11]. The exact molecular events underlying tau toxicity are so far unknown. It is also unclear whether tau’s microtubule-related activities are involved in such a mechanism and whether they are preceding the formation of tau fibrils.

The development of tau pathology is accompanied by post-translational modifications (PTMs) of tau with increased phosphorylation (hyperphosphorylation) at selected sites being the most studied one [12, 13]. Practically all of the phosphorylation sites that have been identified in tau from AD patients result in a decreased interaction of tau with microtubules, which makes it unlikely that phosphorylated tau exerts a direct gain of function effect on microtubule-related activities. Tau is also subjected to proteolytic cleavage, which may affect microtubule-related activities. In fact, a systematic analysis of tau deletion constructs has revealed regions in tau’s carboxy-terminal tail, which have a large effect on modulating tau’s interaction with microtubules, both in a positive and negative manner [14].

Microtubules are the primary interaction partner of tau and at physiological conditions more than 80% of tau is bound to them [15]. Tau binds to microtubules through a flexible array of distributed weak sites located in tau’s microtubule-binding region (MBR) [16]. Interactions with microtubules have also been identified in the C-terminal tail domain, which flanks tau’s MBR [17]. Tau’s interaction with microtubules is highly dynamic and occurs in the densely packed axonal microtubule array via a kiss-and-hop mechanism with a dwell time on a single microtubule-binding site in the millisecond range [18]. The dynamics of the tau-microtubule interaction explains why, under physiological conditions, tau does not negatively affect microtubule-dependent transport despite the high degree of occupation at sites that overlap with kinesin-binding [19]. On the other hand, this implies that changes in the interaction of tau with microtubules, in particular changes which reduce the dynamicity of the interaction and increase tau’s dwell time on a microtubule-binding site have the potential to adversely affect vital microtubule-dependent processes such as the transport of vesicles or mitochondria. In this regard, proteolytic cleavage of tau at the C-terminus could affect the physiological interaction of tau with microtubules.

Proteolytic cleavage of tau’s carboxy-terminal tail has been observed at Asp421 generating a tau species lacking the last 20 amino acids [20]. Cleavage is performed by caspase-3, a member of a group of cysteine proteases that cleave after specific aspartate residues. Caspase-3 activation is induced in apoptotic processes but has also non-apoptotic functions [21]. In fact, caspase-3 is activated in the brain of aged monkeys [22] and both, activated caspase-3 and caspase-3-cleaved tau, were detected in the forebrain of aged mice [23]. Increased activated caspase-3 and accumulation of caspase-3-cleaved tau were also present in an animal model of traumatic brain injury [24] and truncation of tau at the caspase-3 cleavage site was associated with neurodegeneration and tangle formation in animal models of AD [25]. Caspase-3 cleaved tau was also observed in the forebrain of patients with AD and Tauopathic Frontotemporal Dementia [26]. However, despite this correlation, it is still unclear how cleaved tau impairs the function of neurons. It is obvious that a better understanding of the cellular and molecular events on the path of tau-dependent neurodegeneration are also crucial for the development of novel mechanism-based therapeutic interventions.

To determine the extent and influence of tau cleavage at the caspase-3 site, we performed immunoblot analysis of mouse hippocampi and temporal neocortex from AD patients. We determined changes in the dynamics of the tau-microtubule interaction using fluorescence decay after photoactivation (FADP) experiments and single-molecule tracking (SMT) of tau in model neurons. We investigated the influence of tau cleavage on axonal transport by tracking mitochondria and single APP vesicles and scrutinized the effect of microtubule-modulating drugs on binding dynamics and transport parameters. The results reveal a novel gain of function mechanism through which a PTM of tau induces changes in the dynamics of the tau-microtubule interaction that cause axonal transport defects and neuronal degeneration before potentially toxic tau oligomers are formed. The data also show that drugs that target microtubules and affect microtubule structure have the potential to positively modulate deleterious changes in the tau-microtubule interaction.

## Material and methods

### Materials

Chemicals, cell culture media, supplements, culture flasks, plates, dishes and other plastic material were obtained from Sarstedt (Nümbrecht, Germany), Sigma-Aldrich (Deisenhofen, Germany) and Thermo Fisher Scientific (Waltham, USA), unless otherwise stated. Epothilone D (EpoD) was a kind gift from Amos Smith 3rd (University of Pennsylvania) and was prepared as previously described [27, 28]. The spectroscopic properties of the compound were identical to those reported in the literature. Compound purity was >95% as determined by LC-MS and NMR analyses.

### Animals

All animals were kept and killed in accordance with the German animal care regulations based on the FELASA guidelines. C57Bl/6J mice (Envigo, Netherlands) of 2 months and 27 months were used to prepare the brains of mice. TAU^−/−^ (tau knockout) mice kept on a C57BL/6J background [29] were used to generate primary neurons and organotypic hippocampal slices.

### Human material

Temporal cortex brain tissue of 7 AD patients (4 female, 3 male) and 6 non-demented controls (5 female, 1 male) dying without any history of neurological or psychiatric illness was used. Case recruitment, autopsy and data handling have been performed in accordance with the ethical standards as laid down in the 1964 Declaration of Helsinki and its later amendments as well as with the convention of the Council of Europe on Human Rights and Biomedicine and had been approved by the responsible Ethics Committee of Leipzig University (GZ 01GI9999-01GI0299; Approval # 282–02). Informed consent was obtained from all subjects or their legal representatives. All cases were neuropathologically assessed for NFT stage according to [30] and for Aß/amyloid plaque score according to [31]. The mean age of the AD cohort (80.1 ± 7.0 years) was not significantly different from the controls (76.3 ± 6.7 years). The mean Braak stage of the AD patients was 3.9 ± 0.4 and for controls 2.5 ± 1.2. The postmortem interval of the AD cohort (73 h ± 37 h) has been insignificantly longer than that of controls (54 ± 37 h).

### Construction of expression vectors and virus preparations

Tau variants produced herein were based on human full-length tau (hTau441wt) equipped with a N-terminal PAGFP tag, which was constructed previously in a pRc/cytomegalovirus (CMV) expression vector (Life Technologies, USA)[32]. The Halo-tagged full-length tau variant encoded in plasmid pJJH1440 has been described in [18]. A truncated tau corresponding to the caspase-3 cleavage product (TauC3) was generated by insertion of a stop codon (TAG) after position Asp421. For this purpose, a plasmid with the tau441 sequence inserted into pJJH1308 [33] was used to first substitute the SfiI/NheI fragment for the PCR-generated truncated version to yield pJJH1579 (mCherry-hTauΔ421), which was then subcloned into pJJH1440 (Halo-hTau441wt; [18]) to give pJJH1581 (Halo-hTauΔ421). The mCherry tag in pJJH1579 was substituted by a PCR-generated PAGFP sequence by in vivo recombination in yeast to yield pJJH1583 (PAGFP-hTauΔ421). Sindbis virus expression plasmids were based on the yeast/*E. coli*/neuronal triple shuttle vector pJJH1260 [33]. The mCherry-tagged full-length tau441wt sequence was inserted under the control of the Psg promoter, again by in vivo recombination with a PCR-generated fragment (pJJH2254; Sinrep5-mCherry-hTau441wt). The truncated version was obtained by another in vivo recombination with a custom-made oligonucleotide (Biolegio, Nijmegen, Netherlands), substituting the C-terminal tau-coding sequence for two consecutive stop codons (TGA TAA) followed by a BamHI recognition sequence after the codon for Asp421 (pJJH2683, Sinrep5-mCherry-hTauΔ421). Complete sequences of all final constructs can be obtained upon request. Both Lentivirus and Sindbis virus particles were produced as described [33]. Expression plasmid for PAGFP-α-Tubulin was created as described earlier [18]. pEGFP-n1-APP was obtained from Zita Balklava and Thomas Wassmer (Addgene plasmid # 69924; http://n2t.net/addgene:69924; RRID: Addgene_69924). Succinate dehydrogenase complex subunit D (SDHD)-eGFP was a kind gift from Prof. Karin Busch (University of Münster).

### Cell culture, transfections and infections

PC12 cells were cultured in serum-DMEM as previously described [34]. The cell line had been negatively tested for mycoplasma contamination and had been authenticated by “Staatliches Gewerbeaufsichtsamt Hildesheim, Zentrale Unterstützungsstelle Abfall, Gentechnik und Gerätesicherheit” by STR profiling. For tubulin polymerization assays or tracking experiments, constitutively mCherry-Tau or -TauC3-expressing cells were produced by infection with lentiviral particles that carry the respective construct. The cells were amplified and frozen for later transfections with PAGFP-tubulin, SDHD-eGFP or pEGFP-n1-APP. The transduced cells expressed ~10^7^ molecules of mCherry-Tau or -TauC3 molecules per cell as assessed by immunoblot analysis of cell lysates (Supplementary Fig. 1A), corresponding to a concentration of 10 µM in the cells (assuming an average cell volume of 2000 µm^3^ for a mammalian cell line; [35]). Endogenous tau in differentiated PC12 cells has been calculated to be 3–4 μM [36], therefore it can be estimated that overexpressed human tau is approximately three-fold the amount of endogenous tau. All transfections of PC12 cells were carried out with Lipofectamine 2000 (Thermo Fisher Scientific, USA) as previously described [34].

For the shRNA mediated knockdown of tau, lentiviral particles containing three target-specific constructs that encode 19–25 nt (plus hairpin) shRNA designed to knockdown tau gene expression (sc-61900-V; Santa Cruz Biotechnology, Inc.) were added to the culture. After overnight exposure, the medium was replaced with fresh serum-DMEM. Five days after infection, shRNA-expressing cells were selected with 5 μg/ml puromycin dihydrochloride (Santa Cruz Biotechnology, Inc.). The medium was changed every 2–3 days and the culture continued for at least 12 days. To determine the tau expression, cells were differentiated neuronally for 5 days in DMEM with 1% (vol/vol) serum containing 100 ng/ml 7S mouse NGF (Alomone Laboratories, Germany) and used for immunoblot analysis.

Primary cortical neurons from tau knockout mice were prepared from cerebral cortices of mouse embryos (days 14–16 of gestation) as previously described [33]. The cells were plated out at 2.5 × 10^4^ cells/cm^2^ on poly-L-lysine- and laminin-coated coverslips and infected with lentivirus initiating expression of Halo-tagged TauC3.

### Immunoblot analysis

PC12 cell lysates were prepared by scraping off the cells from a tissue culture plate with ice-cold RIPA buffer (50 mM Tris-HCl, 150 mM NaCl, 1 mM EDTA, 1% NP-40, 0.5% sodium deoxycholate, and 0.1% SDS, pH 8.0) in the presence of protease and phosphatase inhibitors (1 mM PMSF, 10 mg/ml each of leupeptin and pepstatin, 1 mM EGTA, 1 mM sodium orthovanadate, 20 mM sodium fluoride, and 1 mM sodium pyrophosphate). Cell suspensions were incubated on ice for 30 min with shaking and cleared by centrifugation at 13,000 × *g* at 4 °C for 10 min.

Brain tissue was homogenized in 4 ml of lysing solution per gram of brain, sonicated (10–15 pulses), and centrifuged for 10 min at 13,000 × *g* at 4 °C. The supernatant was frozen and stored at −80 °C. Protein concentration was determined using a bicinchoninic acid protein assay kit (Thermo Fisher Scientific, USA). The samples were subjected to SDS–PAGE and transferred to Immobilon-P membranes (Millipore, USA), followed by immunoblotting. The following antibodies were used for detection: anti-GAPDH (AB2302, Millipore), TAU-5 (556319, BD Pharmingen), panTau (A0024, Dako), TauC3 (AHB0061, Thermo Scientific), and peroxidase-conjugated donkey anti-mouse (Jackson ImmunoResearch Laboratories, Inc., USA) or goat anti-rabbit (EMD Millipore Corp. MA, USA) secondary antibodies.

Protein bands were detected using enhanced chemiluminescence with SuperSignal West Dura extended duration substrate (Thermo Fisher Scientific, USA) according to the manufacturer’s protocol. Quantification of the blots was carried out with Gel-Pro Analyzer 4.0 (Media Cybernetics L.P., USA) or with FusionCapt Advance (Vilber Lourmat, France). To normalize the signal against the loaded samples, the membranes were stained for 5 min in 0.1% Coomassie R250 in 50% methanol, 10% acetic acid, followed by a differentiation of the staining in 50% methanol, 7% acetic acid.

For the immunoblot analysis, the investigator was blinded to the group allocation during the experiment.

### Organotypic hippocampal slice culture

Organotypic hippocampal slice cultures were prepared from 7-day-old mouse pups of tau knockout mice on a C57BL/6J background strain and processed as previously described [37, 38]. Briefly, brains were removed from skulls, hippocampi were isolated from both hemispheres and cut using a tissue McIllwain chopper (400 µm thickness). Properly shaped slices were selected and transferred onto membrane inserts (Merck Chemicals GmbH, Germany) with fresh culture medium (MEM, pH 7.18 supplemented with 25% horse serum, 25% Basal Medium Eagle, 1% glutamine, 0.6% glucose, 0.5% Pen-Strep). Tissue cultures were kept at 37 °C and 5% CO_2_. At 11 days in vitro (DIV), the culture medium was exchanged for supplemented Neurobasal medium (1% glutamine, 1% N1 supplement, 0.6% glucose, 0.5% horse serum, 0.5% Pen-Strep). At 12 DIV, the slices were infected with Sindbis virus by the droplet method in order to induce the expression of mCherry which was fused to either human Tau441wt or TauC3. The cultures were fixed with ice-cold fixing solution (4% paraformaldehyde, 4% sucrose in PBS) at 15 DIV and mounted with Confocal Matrix (Micro-Tech-lab, Austria).

### Confocal imaging of dendritic segments and neuronal arbors

Confocal images were acquired using a Nikon Eclipse TE2000-U inverted microscope (Nikon, Japan) equipped with a ×60 oil-immersion objective (NA, 1.4) and a C1 confocal laser scanning unit and EZ-C1 software. Dendritic segments (>25 µm) were captured using a helium/neon 543-nm laser from secondary and tertiary branches of the apical arbor of Cornu Ammonis 1 (CA1) pyramidal neurons. Micrographs (voxel size: 0.06 × 0.06 × 0.30 µm) were blind deconvolved using AutoQuantX vX3.0.5 software (Media Cybernetics Inc., USA) in order to improve the signal-to-noise ratio, and the analysis of individual dendritic spines was carried out using the morphometric software 3DMA v0204 [39]. The spines were classified into mushroom, stubby and thin based on the ratio of head to neck diameter and the ratio of length to neck diameter as described before [40, 41]. Confocal tiles of complete CA1 hippocampal arbors were acquired as previously described [42] using a Zeiss 510 META NLO confocal laser scanning microscope (Zeiss, Oberkochen, Germany) equipped with a ×40 oil-immersion objective (NA, 1.3) and a Helium/Neon 543 nm laser governed by LSM 510 v4.0 SP2 software. Overlapping tiles (7–13 per neuron; voxel size: 0.3 × 0.3 × 0.45 µm) were deconvolved via Huygens Remote Manager v3.5 software (Scientific Volume Imaging B.V., Hilversum, Netherlands). The deconvolution was carried out using a theoretical PSF and employing a classical maximum likelihood estimation as deconvolution algorithm with 15 iterations and a quality criterion of 0.05. The overlapping tiles were manually stitched together using VIAS v2.2 software (Mt. Sinai School of Medicine, USA). The neuronal 3D reconstruction of apical and basal arbors was performed by Neuromantic software (University of Reading, UK) in semi-automatic mode.

### Live-cell imaging for photoactivation and axonal transport experiments

For FDAP and transport experiments, wild-type, constitutively mCherry-Tau or TauC3 expressing, or lentivirally transduced PC12 cells expressing shRNA for tau knockdown were plated on 35-mm poly-L-lysine and collagen-coated glass-bottom culture dishes (MatTek, USA). After transfection, PC12 cells were neuronally differentiated by medium exchange for DMEM with 1% (vol/vol) serum containing 100 ng/ml 7S mouse NGF (Alomone Laboratories, Germany). Cultivation was continued for 4 days with medium exchange for DMEM with reduced serum containing NGF and without phenol red 1 day prior to live imaging. Live imaging of PC12 cells for photoactivation experiments was performed using a laser scanning microscope (Eclipse TE2000-U inverted and Nikon Eclipse Ti2-E (Nikon, Japan) equipped with a LU-N4 laser unit with 488-nm and 405-nm lasers and a Fluor 60× ultraviolet-corrected objective lens (NA 1.4) enclosed in an incubation chamber maintaining 37 °C and 5% CO_2_. Automated image acquisition of PAGFP-tau or -α-tubulin expressing cells after photoactivation was essentially performed as described previously [43]. Briefly, photoactivation of a 6 μm long neurite segment was performed with a 405-nm laser. A set of consecutive image series (time stack) was obtained at a frequency of 1 frame/s, and 112 frames were collected per activated cell at a resolution of 256 × 256 pixels. The same microscope was used to monitor the transport of SDHD-eGFP labeled mitochondria in PC12 cells. Lentiviral expression of the respective tau background was confirmed by visualizing the mCherry-Tau expression using a 561 nm laser. Time series of 8 min were acquired using a timestep of 2 s between each frame at a resolution of 512 × 512 pixel (pixel size 98 nm) using a 488-nm laser. Live imaging for tracking analysis of APP transport in PC12 cells was performed using a Zeiss Cell Observer Z1 (Zeiss, Germany) with dual-color high-speed confocal imaging using the CSU-X1 spinning disc technology from Yokogawa equipped with a 488-nm optically pumped semiconductor laser and a 561-nm laser diode enclosed by a sample incubation chamber controlled by a Zeiss TempModule S1. pH-stability was maintained by addition of 30 mM HEPES buffer to the cell culture medium prior to image acquisition. For image acquisition an Alpha Plan-Apochromat ×63 (NA 1.46) objective was used. After confirmation of mCherry-tau expression using the 561-nm laser, an image series (time stack) of 60 s was acquired at a speed of five frames per second captured with a Hamamatsu ORCA flash V3 and a 2 × 2 binning at a resolution of 640 × 640 pixels (pixel size 172 nm) using the 488-nm laser only. Image series were deconvoluted via Huygens Remote Manager v3.5 software (Scientific Volume Imaging B.V., Hilversum, Netherlands) using a theoretical PSF and employing a classical maximum likelihood estimation as deconvolution algorithm with 20 iterations and a quality criterion of 0.05.

### FDAP data analysis

Effective diffusion constants were obtained by fitting the fluorescence decay data from photoactivation experiments using a one-dimensional diffusion model function for FDAP, as previously described [15]. A reaction-diffusion model was used as described previously [43] to estimate the association rate k*_on_ and the dissociation rate k_off_ constant of tau or tubulin binding. Processing and analysis of individual FDAP curves was performed as previously described [14] using a custom C-based tool called cFDAP. The fitting procedure was used to obtain k*_on_ and k_off_ from every single FDAP curve, while the *χ*^2^ value was used as an indicator of the goodness of fit of the model function.

### Tracking and quantification of axonal transport

Mitochondrial transport was tracked with the Fiji plugin TrackMate [44]. A general distinction between mobile and stationary mitochondria was made when their total motion was greater than their diameter. These estimates were based on the idea that the diameter could be measured accurately. However, since the diameter of the mitochondria changed from frame to frame due to movement in *z*-direction, rotation or fusion and fission events, a distribution of all recorded diameters of each track was compared with a one-sample *t*-test against the displacement of the track. The *t*-test resulted in two values: the *t* and the *p* value. The *t* value was used to distinguish which side of the distribution the shift was on, i.e., if the displacement was smaller or larger than the mean diameter. On the other hand, the *p* value was used to estimate whether a mitochondrion was significantly larger or smaller than the mean diameter value. All identified objects that did not differ significantly from the diameter distribution were regarded as undefined and excluded from further analysis. This led to the following definition of fractions for mitochondrial transport: mobile *p* > 0.95 and *t* < 0, stationary *p* > 0.95 and *t* ≥ 0 and undefined when *p* ≤ 0.95.

The vesicle transport of eGFP labeled APP-vesicles within axon-like processes of PC12 cells was tracked and analyzed using Imaris v 9.2 (Bitplane, Oxford Instruments). Vesicles were detected by the Gaussian filtered intensity of their signal within a diameter of 500 nm surrounding the respective signal peak. Vesicle transport was tracked by an autoregressive motion algorithm with a maximum distance of a future position of the signal of 0.5 µm and a maximum gap size of 2 frames (400 ms). A reference point at the axon hillock was used to determine the direction of the tracked vesicles, while only the tracks that extended over a period of at least 3 s (15 frames) were considered for further analysis. Quantified transport parameters were exported to spreadsheets (Excel, Microsoft Corporation, USA) and processed further using TIBCO Spotfire Data Analysis Software (TIBCO Software Inc., USA). Vesicles which exceeded a displacement of more than 0.75 µm over the observation time of 60 s were designated as mobile, otherwise regarded as stationary. For further analysis of individual trajectories of mobile vesicle with respect to changes in state, we employed the localization data provided by the spot (vesicle) detection and autoregressive motion algorithm of Imaris. Movement along the neurite was assessed by the localization of a vesicle frame by frame. The displacement was calculated as a difference between two successive locations of a vesicle along the neurite and normalized by the time between these points. Thus, a gap in the localization of a vesicle for 1 or 2 frames was accounted for. To define a shift in either retrograde or anterograde direction, a threshold was set at 0.02 µm/frame, which is the average minimum speed (about 0.1 µm/s) based on the analysis by the autoregressive motion algorithm and calculations for the control experiments (*n* = 21 cells, 470 mobile vesicles in total total). A displacement for less than 0.02 µm/frame was assigned to be a stalling event, while a displacement for more than 0.02 µm/frame was assigned to be anterograde or retrograde movement, respectively. The corresponding R scripts used for this analysis are available online on GitHub (https://github.com/Department-of-Neurobiology/Tau421_transport_analysis).

### Single-molecule microscopy, localization, tracking and determination of residence time

For single-molecule imaging of tau, PC12 cells and primary cortical neurons were cultured as previously described [18]. Prior to imaging, cells were labeled substoichiometrically by incubation with serum-DMEM containing tetramethylrhodamine (TMR) HaloTag ligand for 15 min at 37 °C. The labeling efficiency corresponded to a stochiometry of <0.02% of Halo-tau. After several washes with serum-DMEM without phenol red and a short recovery time, the cells were subjected to single-molecule imaging using an Olympus excellence cell TIRF microscope (total internal reflection fluorescence), which was equipped with 561-nm laser (200-mW; Olympus, Japan) and a back-illuminated electron-multiplied charge-coupled device camera (C9100-13; Hamamatsu, Japan) in HILO mode (highly inclined and laminated optical sheet). An objective with ×150 magnification NA 1.45 (UAPON ×150/1.45; Olympus) was used for the HILO illumination. A digital complementary metal–oxide–semiconductor camera (ORCA-Flash4.0 V2 C11440-22CU; Hamamatsu) was used for rapid single-molecule tracking. The light emitted from the sample was filtered using a quad-band bandpass filter (FF01446/523/600/677; Semrock, Rochester, NY). Imaging of living cells was made possible by enclosing the microscope with an incubation chamber that maintained 37 °C and 5% CO_2_ (Olympus-PeCon). Localization and trajectory reconstruction were carried out as previously described [18]. The theoretical localization precision of Halo-tau was 16.7 ± 0.2 nm. For further analysis, a mask was placed over the cell processes and trajectories outside this mask were removed. The determination of the residence time of tau in the MT-bound state was achieved by counting how many consecutive frames a single localized signal stayed within a radius of 50 nm. The respective frequency histograms were fitted by a single exponential function to estimate the residence time.

### Statistical analysis

Statistical analysis was carried out with SPSS v26 (IBM, USA) and GraphPad Prism v8.0.1 (GraphPad Software, USA). All data sets were tested for normality using D’Agostino-Pearson and Shapiro–Wilk test. If necessary, data sets were log transformed in order to enable further statistic testing. Statistical outliers were identified using the ROUT method. The homogeneity was assessed using Levene’s test. An unpaired two-tailed *t*-test was used to compare two data sets. In the event of unequal variances, the Welch’s correction was applied. A one-way ANOVA was performed to compare more than two data sets. To determine the effect influenced by two factors, a two-way ANOVA was performed, both types of ANOVA followed by post hoc Fisher LSD test. All statistical values are expressed as mean ± SEM.

## Results

### The ratio of caspase-3-cleaved tau (TauC3) doubles in the hippocampus of senescent mice

**Fig. 1:**
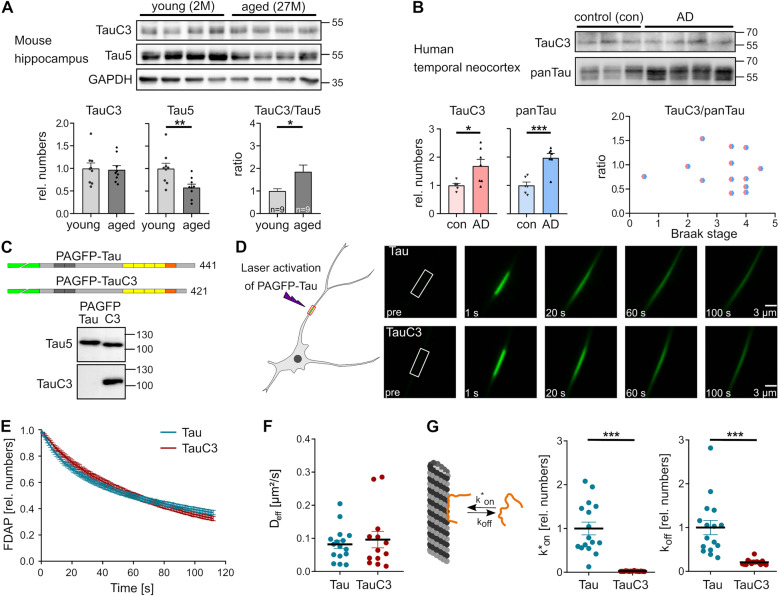
Caspase-3-cleaved tau (TauC3) is present in mice and patients and shows decreased dynamics of its microtubule (MT) interaction. **A** Immunoblots from lysates of hippocampal brain tissue from young (2 months (M)) and aged mice (27 M) showing caspase-3-cleaved tau (TauC3), total tau (Tau5) and GAPDH as loading control. Molecular mass standards are indicated. The relative amounts of TauC3/GAPDH, Tau5/GAPDH, and TauC3/Tau5 are shown below (mean ± SEM; *n* = 9). **B** Immunoblots from lysates of the human temporal neocortex from healthy controls (con) and AD patients (AD) showing TauC3 and total tau (panTau). Molecular mass standards are indicated. The relative amounts of TauC3/Coomassie, panTau/Coomassie and TauC3/panTau with respect to the Braak stage are shown below (mean ± SEM; *n* = 6 (con) and *n* = 7 (AD)). **C** Schematic representation of the expressed tau constructs. The MT-binding repeat regions (RR1–RR4) are indicated by yellow boxes, the pseudorepeat region in orange and the N-terminal PAGFP-fusion in green. Adult-specific exons in the N-terminus of tau (N1, N2) are shown in dark gray. Immunoblots of PC12 lysates after transfection with the respective tau constructs are displayed below, which shows the specific detection of the shorter tau construct with the TauC3 antibody. Molecular mass standards are given. **D** A schematic representation of an FDAP experiment is shown on the left. Representative time-lapse micrographs of fluorescence decay after photoactivation (FDAP) in an axon-like process are shown on the right. A 6 µm long segment (white box) in the middle of a process was photoactivated and the fluorescence decay over time within this region was monitored. **E** FDAP diagrams after photoactivation of PAGFP-Tau (Tau) and PAGFP-TauC3 (TauC3)-expressing cells. Mean ± SEM of 16 (Tau) and 15 (TauC3) cells is shown. Scatter plots of the effective diffusion constants (D_eff_) (**F**), and association (k*_on_) and dissociation rate constants (k_off_) (**G**) (mean ± SEM, *n* = 16 (Tau) and *n* = 15 (TauC3)). A schematic representation indicating k*_on_ and k_off_ of the MT-Tau interaction is shown in **G**. Statistically significant differences between samples determined by an unpaired Student’s *t* test are indicated. *p < 0.05; **p < 0.01; ***p < 0.001.

The hippocampus is a highly vulnerable brain region and is among the first that is affected during aging. Thus, we examined whether caspase-3-cleaved tau (TauC3) is present in the hippocampus of mice and whether its amount changes during aging. Western blot analysis showed that the total amount of tau was significantly lower in senescent mice relative to a control protein (GAPDH) (Fig. 1A). In contrast, TauC3 as detected with an antibody that specifically recognizes tau when truncated at Asp421 [20, 45] remained constant, which resulted in an about two-fold higher ratio of TauC3 to total tau in senescent compared to young mice (*p* < 0.05). Calibration relative to lysates of cells expressing full-length tau or TauC3 revealed that TauC3 was ~19% of the total tau in the hippocampus of senescent mice.

We also examined lysates from the temporal neocortex from AD patients and non-demented controls, a region which degenerates during disease progression. For the TauC3 analysis in the human brain, we chose temporal cortex tissue compared to the hippocampus due to the greater homogeneity and the lower sample variability. In addition, the tau pathology correlates better with disease-relevant higher Braak stages (above Braak stage IV) in the temporal cortex tissue. Both, the amount of total tau and TauC3 were significantly higher in samples from AD brain compared to controls (Fig. 1B). We did not observe any change in the ratio of TauC3 to total tau with respect to different Braak stages used to classify the degree of pathology in AD. Therefore, the data suggest that TauC3 production is influenced by senescence- rather than disease-related processes and that tau cleavage can thereby modulate the physiological function of nerve cells during aging.

### TauC3 shows reduced dynamics of its microtubule interaction

During physiological conditions, tau interacts with neuronal microtubules in a highly dynamic fashion. We used FDAP experiments to determine a potential change in the dynamics of the interaction of full-length tau and TauC3 with microtubules in axon-like processes of model neurons. All transduced or transfected tau constructs were based on the human tau sequence, since mouse and human tau differ in their sequence, especially at the N-terminal end, which can influence its interaction with other proteins [46, 47]. The constructs were N-terminally tagged with photoactivatable GFP (PAGFP) and exogenously expressed in PC12 cells that were differentiated to a neuronal phenotype. Western blot analysis confirmed that PAGFP-TauC3 is expressed similarly to full-length tau and is selectively recognized by the TauC3-specific antibody (Fig. 1C). After focal activation, both constructs showed a similar dissipation from the region of activation (Fig. 1D). However, the FDAP curves revealed a different decay indicating different dynamics of the interaction of TauC3 with microtubules compared to full-length tau (Fig. 1E). Calculation of the effective diffusion constants [15] yielded similar values for full-length tau and TauC3 (Fig. 1F) indicating that the cleavage did not affect the binding equilibrium between free and microtubule-bound tau. However, application of a previously developed refined reaction-diffusion model of the tau-microtubule interaction [43] showed that the binding constants (k*_on_ and k_off_ rates) were drastically reduced for TauC3 compared to full-length tau (Fig. 1G). The results show that carboxy-terminal cleavage of tau at the caspase-3 site strongly reduces the dynamicity of the tau-microtubule interaction in axon-like processes.

### The residence time of individual TauC3 molecules is increased on axonal microtubules

**Fig. 2:**
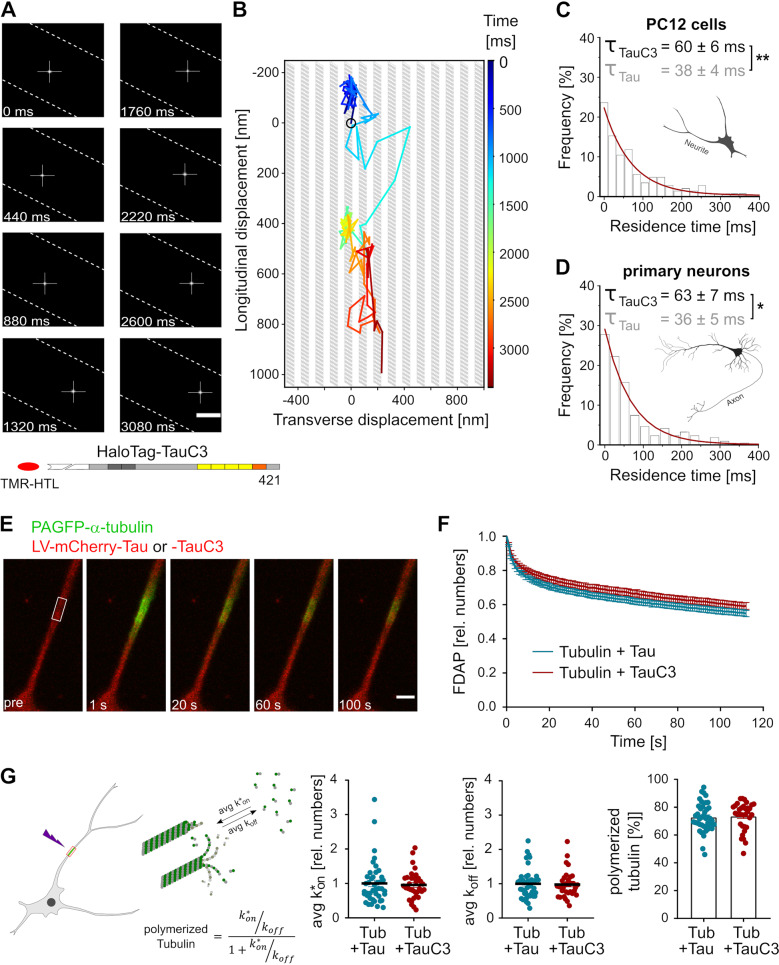
The residence time of individual TauC3 molecules is increased on axonal microtubules without modifying axonal microtubule polymerization. **A** Single-molecule tracking of a single TauC3 molecule in an axon-like process of a differentiated PC12 cell. The position of tau at selected times is displayed; dashed lines indicate the limit of the neurite. TauC3 was fused N-terminally with a HaloTag to enable substoichiometric labeling with TMR-HTL as indicated below. Scale bar, 0.5 μm. **B** Pseudotrajectory generated from the time series shown in **A** (3.4 s = 156 frames) indicating fast and undirected movement in the longitudinal and transverse direction of the axonal process. For comparison, the thickness and density of MTs are indicated schematically by gray bars. The starting point is indicated by a black circle and the time is color-coded as shown on the right. Representative histograms of the residence times of Halo-TauC3 on MTs in PC12 cells (**C**) and in primary neurons prepared from tau knockout mice (**D**). The monoexponential fit is indicated by a red line. The time constant (residence time) is given above (mean ± SEM for *n* = 8 (PC12 cells) and *n* = 5 (primary neurons) from 2413 and 1410 temporally immobile molecules, respectively). The residence time of full-length tau was previously determined (Janning et al. [18]) and is shown in gray. **E** Representative micrographs of FDAP time-lapse recordings of PAGFP-α-tubulin in a process of a PC12 cell. mCherry-tagged Tau or TauC3 was co-expressed by lentiviral (LV) expression. The photoactivated segment is indicated by a white box. Scale bar, 5 µm. **F** FDAP diagrams of PAGFP-α-tubulin in cells co-expressing mCherry-Tau (Tau) or mCherry-TauC3 (TauC3). Mean ± SEM of 42 cells for Tau and 34 cells for TauC3 is shown. **G** Scatter plots of average association (avg k*_on_), average dissociation (avg k_off_) rate constants with mean ± SEM and percent of polymerized tubulin (*n* = 42 and 34 cells for Tau and TauC3, respectively). A schematic representation is shown on the left which gives avg k*_on_ and avg k_off_ of the tubulin-MT equilibrium and the calculation of polymerized tubulin. All transfected tau constructs are based on the human tau sequence. Statistically significant differences determined by unpaired Student’s *t* test are indicated. *p < 0.05; **p < 0.01.

We used SMT to visualize the interaction of individual tau molecules in axon-like processes in order to directly determine the residence time of tau on a single microtubule-binding site. A TauC3 construct with an N-terminal fusion of HaloTag was expressed in the cells, substoichiometrically labeled with TMR-HTL and imaged by TIRF-microscopy in the HILO mode with a high-speed camera (Fig. 2A). A typical trajectory shows fast, longitudinal and transversal displacements of a fluorescent tau molecule in the cellular process indicating rapid movement between binding sites that were several 100 nanometers apart (Fig. 2B). The movement corresponded to the same kiss-and-hop interaction that we previously described for full-length tau [18]. The frequency histogram of residence times was fitted to a single exponential decay and yielded a mean dwell time of tau on a single microtubule of about 60 ms, which was ~60% higher than the dwell time of full-length tau (Fig. 2C). The mean dwell time was similarly increased (~75%) in primary neurons prepared from tau knockout animals, which were lentivirally transduced with a HaloTag-TauC3 construct (Fig. 2D). The results confirm that carboxy-terminal cleavage of tau reduces the dynamicity of the tau-microtubule interaction by significantly increasing the time that tau dwells on a single microtubule-binding site before it hops to the next one.

Tau is a microtubule-associated protein that regulates the tubulin-microtubule balance in the axon. To determine the influence of TauC3 on microtubule dynamics we co-expressed mCherry-tagged full-length tau or TauC3 together with PAGFP-tagged α-tubulin (PAGFP-α-tubulin) in neuronally differentiated cells. Changes in MT dynamics were then monitored by FDAP-measurements from cells in which the fluorescence of PAGFP had been focally activated in the middle of a process (Fig. 2E). FDAP curves were very similar in cells expressing full-length tau or TauC3 (Fig. 2F). To determine the fraction of free and bound tubulin, we fitted the FDAP curves with a reaction-diffusion model. Indeed, the amount of polymerized tubulin was ~70% and did not differ significantly between cells expressing full-length tau or TauC3. Also, application of the reaction-diffusion model yielded k*_on_ and k_off_ values for average microtubule polymerization which were very similar in cells expressing the two different constructs (Fig. 2G). The results show that the cleavage of tau at the caspase-3 site does not affect the axonal tubulin-microtubule balance, despite influencing the dynamics of its interaction with microtubules.

### TauC3 slows the microtubule-dependent transport of mitochondria and reduces the processivity of APP-vesicle transport

**Fig. 3:**
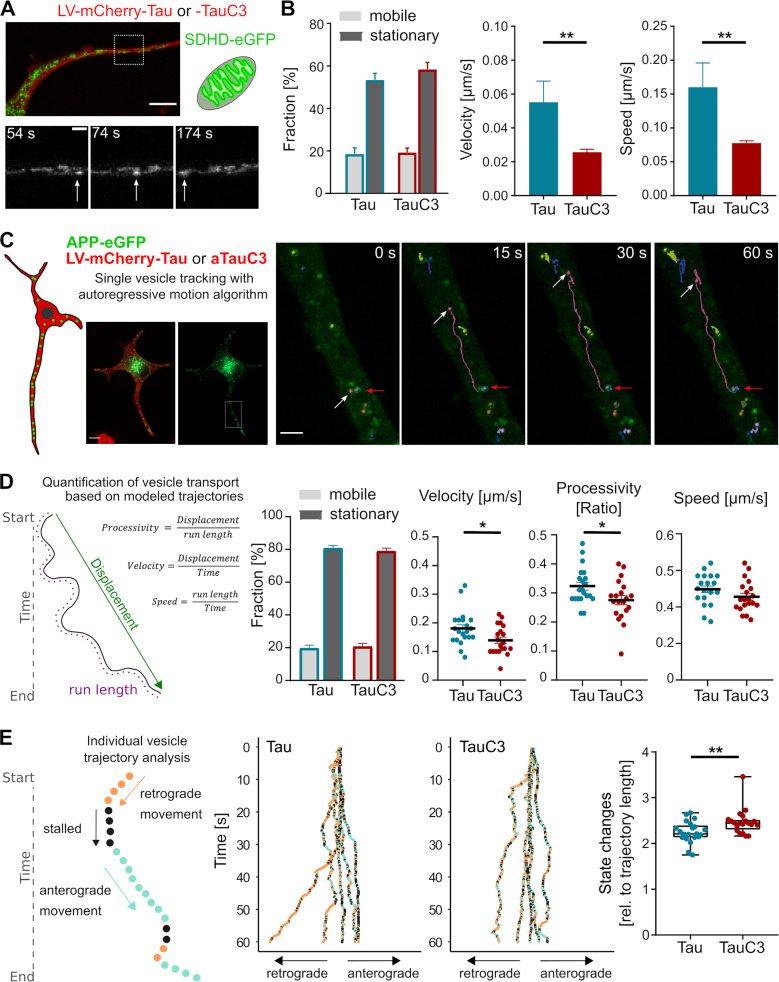
TauC3 slows down the microtubule-dependent transport of mitochondria and reduces the processivity of APP-vesicle transport. **A** Micrograph of a PC12 cell expressing eGFP-SDHD for tracking the movement of mitochondria in living cells. mCherry-tagged Tau or TauC3 was co-expressed by lentiviral (LV) expression. A moving mitochondrion is indicated by an arrow in the enlarged time-lapse images shown below. Scale bar, 10 µm (top) and 2 µm (bottom). **B** Bar graphs showing fractions of stationary and mobile mitochondria in the process. Velocity and speed of the mobile mitochondria are displayed in the middle and right graphs. Mean ± SEM, *n* = 12 cells with 672 trajectories (mCherry-Tau) and *n* = 22 cells with 1137 trajectories (mCherry-TauC3). **C** APP-vesicle tracking in a process of a PC12 cell using eGFP-tagged APP and an autoregressive motion algorithm. mCherry-tagged Tau or TauC3 was co-expressed by lentiviral (LV) expression. The images on the left show an overview of a cell that co-expresses tau and APP. The images on the right show selected points in time of the APP-vesicle movement from the part of the process that is indicated by the white box in the overview image. Arrows point to a moving (white) and a stationary vesicle (red). Scale bars, 10 µm (overview) and 2.5 µm (time lapse). **D** The quantification of the transport parameters is shown on the left. The bar chart shows fractions of mobile and stationary vesicles in the process. The velocity, processivity, and speed of the mobile vesicles are shown in the scatter plots on the right. Each point represents an average value for a respective cell (mean ± SEM of *n* = 21 cells with 470 trajectories (Tau) and *n* = 20 cells with 413 trajectories (TauC3)). **E** The definition of state changes of individual vesicle tracts is shown on the left. The box plot on the right shows the cumulative changes in state. Every dot represents one cell of the data set shown in **D**. The two diagrams in the middle show the phases of movement of 5 long representative trajectories for cells expressing Tau or TauC3. All transduced tau constructs are based on the human tau sequence. Statistically significant differences determined by unpaired Student’s *t* test are indicated. *p < 0.05; **p < 0.01.

If the highly dynamic interaction of tau with microtubules assures that tau does not stand in the way of motor protein-dependent transport, one may expect that reduction of the dynamicity of tau-MT-binding would impair the movement of mitochondria in the axon. Thus, we co-expressed a fluorescence-tagged mitochondrial marker protein (succinate dehydrogenase complex subunit D (SDHD)-eGFP) with mCherry-tagged full-length tau or TauC3 in the cells. The approach made it possible to visualize and track the movement of mitochondria (Fig. 3A). About 20% of mitochondria in axon-like processes were mobile, which is consistent with previous data on mitochondrial transport and docking in axons of cultured neurons [48]. The fraction of mobile mitochondria was similar in full-length tau and TauC3-expressing cells (Fig. 3B, left). Both, velocity and speed of the mobile mitochondria were significantly reduced to about 50% in TauC3-expressing cells compared to cells expressing full-length tau (Fig. 3B, right). The results show that the presence of caspase-3 cleaved tau greatly reduces the velocity of mobile mitochondria in the axon without affecting mitochondrial docking consistent with the hypothesis that a less dynamic tau species acts as a roadblock for mobile mitochondria.

We also examined the influence of tau cleavage on axonal transport of vesicles. We used single vesicle tracking of eGFP-tagged amyloid precursor protein (APP), a key axonal transport cargo [49]. Vesicles were tracked in axon-like processes of cells that had been lentivirally transduced with mCherry-tagged full-length tau or TauC3 (Fig. 3C). About 20% of APP vesicles were mobile in the cells, which is consistent with previous data on transport of APP in cultured neurons [49, 50]. TauC3 expression did not change the fraction of mobile APP vesicles (Fig. 3D, left). To quantify vesicle transport, we determined the effect of TauC3 on velocity, processivity and speed based on the trajectories from each analyzed cell. Both, velocity and processivity of APP-vesicles were significantly reduced in TauC3-expressing cells compared to cells expressing full-length tau. Mean speed was slightly reduced, but the change did not reach significance (Fig. 3D, right). The same was true for the maximum speed on the track, which was slightly lower in the presence of TauC3 (1.45 ± 0.03 µm/s and 1.35 ± 0.04 µm/s (mean ± SEM, *n* = 21 and 23) for full-length tau and TauC3, respectively). Analysis of the individual trajectories revealed a significantly increased number of changes in state (change in direction and transition between movement and arrest) (Fig. 3E). The results indicate that the presence of caspase-3 cleaved tau disrupts axonal transport of APP vesicles by decreasing the processivity of transport and increasing state changes in motion, confirming that a less dynamic tau species is acting as a roadblock for the organelle movement.

### TauC3 induces region-specific dendritic atrophy in CA1 neurons of the hippocampus

**Fig. 4:**
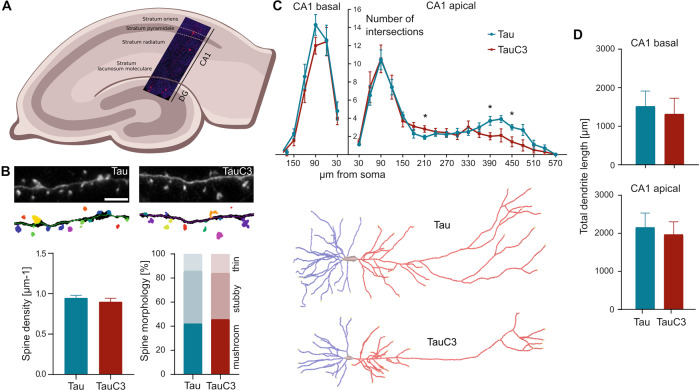
TauC3 induces region-specific dendritic atrophy in CA1 neurons of the hippocampus. **A** Schematic representation of an organotypic hippocampal slice showing neurons in the Cornu Ammonis 1 (CA1) and Dentate Gyrus (DG) region that express mCherry-tagged tau after Sindbis virus infection. The segment was counterstained with DAPI to visualize cell bodies. **B** Representative high-magnification micrographs and corresponding image analysis of apical dendritic segments from CA1 neurons after expression of mCherry-tagged Tau or TauC3 to illustrate the algorithm-based detection of dendritic spines (colored protrusions). Scale bar, 5 µm. Quantification of spine density (left) and spine morphology (right) is shown below (mean ± SEM, *n* = 30 and 34 dendritic segments from 12 and 17 cells (Tau and TauC3, respectively); slices were prepared from 8 and 9 tau knockout animals, respectively). **C** Sholl analysis of apical and basal arbors of CA1 hippocampal pyramidal cells expressing mCherry-tagged Tau or TauC3 (mean ± SEM, *n* = 9–10 cells from 9–10 slices; slices were prepared from 5 and 6 tau knockout animals). Representative reconstructions of the basal (blue) and apical (red) CA1 dendritic arbors are shown below. **D** Quantification of total lengths of basal and apical dendritic arbor (mean ± SEM, *n* = 9–10 reconstructed neurons from 9–10 hippocampal slices per condition; slices were prepared from 5 and 6 tau knockout animals). All transduced tau constructs are based on the human tau sequence. Statistically significant differences determined by unpaired two-tailed *t*-tests are indicated. *p < 0.05.

Damage to axonal transport is an early pathogenic event in AD, which may trigger synaptic changes and dendritic simplification [51–53]. We used virus-mediated expression of tau in organotypic hippocampal slices from tau knockout mice to replace endogenous mouse tau with human full-length tau and TauC3. We focused our analysis on principal neurons of the CA1 region (Fig. 4A) since dendritic extent is largely reduced in CA1 pyramidal neurons of patients with AD [54]. Algorithm-based spine analysis of dendritic segments showed similar spine densities in full-length and TauC3-expressing neurons (Fig. 4B). The fractions of different spine types were also very similar. In contrast, Scholl analysis showed a significantly reduced dendritic complexity in the distal apical tree of CA1 neurons expressing TauC3 compared to full-length tau expressing cells (Fig. 4C). No change was observed in the basal dendritic tree. Total neurite length was slightly reduced in both regions of TauC3-expressing cells compared to full-length tau expressing cultures, but the change did not reach significance (Fig. 4D). The results show that TauC3 induces compartment-specific dendritic simplification in the distal part of the apical dendritic tree. This fits in with the assumption that the regions furthest away from the cell body are affected first by impairment of axonal transport.

### The microtubule-targeting drug Epothilone D normalizes the interaction of TauC3 with microtubules and modulates the transport of APP-vesicles dependent on the presence of overexpressed human tau

**Fig. 5:**
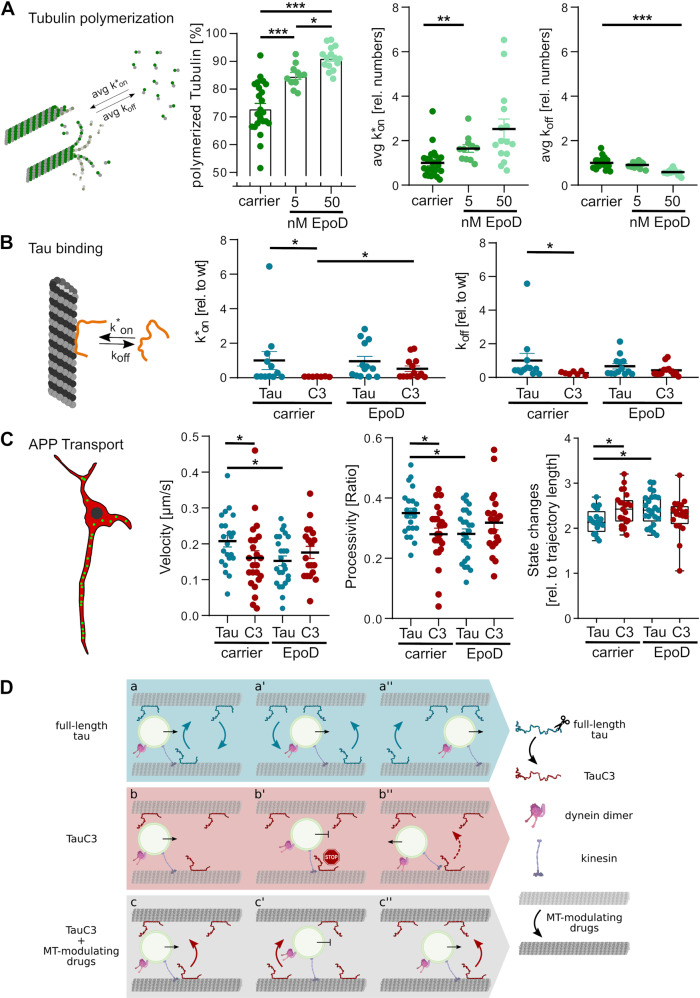
The microtubule-targeting drug Epothilone D increases MT polymer, normalizes the interaction of TauC3 with microtubules, and modulates the transport of APP-vesicles in the presence of overexpressed human tau. **A** Effect of EpoD on the percentage of tubulin polymerized in PC12 cell processes. The average constants for association (avg k*_on_) and dissociation rate (avg k_off_) are shown in the middle and on the right. The rate constants are displayed relative to the carrier control (0.01% DMSO). Mean ± SEM (*n* = 15–24). **B** Effect of EpoD on the association (k*_on_) and dissociation rate constants (k_off_) of the tau-MT interaction in processes of PC12 cells. Rate constants are indicated relative to PAGFP-tau (Tau) in the presence of the carrier control (0.01% DMSO). Mean ± SEM (*n* = 7–13). **C** Effect of EpoD on the transport of mobile APP-vesicles. mCherry-tagged Tau or TauC3 was co-expressed with eGFP-tagged APP and vesicle mobility was determined using an autoregressive motion algorithm. The velocity, processivity, and state changes are shown in the scatter plots. Each point represents an average value for a respective cell (mean ± SEM of *n* = 19–25 cells with 532–624 trajectories). **D** a, a’, a” Time series under physiological conditions: Tau (blue) interacts dynamically with microtubules by kiss-and-hop. Due to the high dynamics of the interaction of tau with microtubules, it does not stand in the way of kinesin-driven vesicle transport. **D** b, b’, b” Time series after caspase-3 cleavage of tau in senescent neurons. TauC3 (red) shows less dynamics in its interaction with microtubules, which can create a temporary roadblock to vesicle movement causing a change in direction. This leads to a reduced processivity of the movement. **D** c, c’, c” Time series after treatment with Epothilone D. EpoD modulates the structure of microtubules, resulting in increased dynamics of the TauC3-MT interaction, similar to full-length tau. However, EpoD also reduces the processivity of APP-vesicle transport in the presence of overexpressed human tau. All transfected or transduced tau constructs are based on the human tau sequence. Treatment with EpoD or carrier control was done 1 h before imaging. Statistically significant differences determined by one-way ANOVA (**A**) or two-way ANOVA (**B**, **C**) and post hoc Fischer LSD are indicated. *p < 0.05; **p < 0.01; ***p < 0.001.

Microtubule stabilizers have structural effects on the microtubule lattice [16] which lead to increased polymerization and may also modulate the interaction of tau with microtubules. Thus, we examined the effect of EpoD, a small-molecule microtubule stabilizer that binds to the taxane site at the luminal surface of microtubules [55], on microtubule polymerization and tau-microtubule interaction. We used FDAP-measurements of cells that had been transfected with PAGFP-α-tubulin to monitor changes in MT dynamics. EpoD increased microtubule polymer in a concentration dependent manner with an increase of ~10% at 5 nM (Fig. 5A, left). Higher concentrations resulted in a further increase in polymerized tubulin. The increase in microtubule polymer was mainly due to an increase in the average k_on_ rate rather than a decrease in the k_off_ (Fig. 5A, middle and right). Despite the change in the amount of microtubule polymer, the binding of full-length tau to microtubules was not affected by the presence of EpoD (Fig. 5B). In contrast, EpoD increased the k_on_ rate of the binding of TauC3 to microtubules and abolished the differences in the k_off_ rates compared to full-length tau. Thus, the data show that EpoD differentially affects the interaction of full-length tau and TauC3 with microtubules. By this, EpoD restored the dynamicity of the interaction of cleaved tau with microtubules close to the level of full-length tau.

If EpoD improves the dynamic interaction of TauC3 with microtubules, one might expect that EpoD also restores axonal transport that is disrupted by the presence of cleaved tau. However, we observed that EpoD did not change vesicle processivity in TauC3-expressing cells or affect the velocity of APP transport and the number of state changes (Fig. 5C). On the other hand, EpoD reduced vesicle processivity in full-length tau expressing cells by ~20%, reduced the velocity of APP transport, and increased the number of state changes to the level of TauC3-expressing cells. This could suggest that EpoD induces structural changes in microtubules which modulate vesicle transport and override the influence of tau dynamics on transport efficiency. To determine whether tau contributes to the modulation of transport by EpoD, we determined whether the effect of EpoD on APP vesicle transport changes in the absence of exogenously expressed human tau and after shRNA mediated knockdown of endogenous rat tau. We observed that, in the absence of exogenous tau, EpoD no longer influences the transport of APP vesicles and that the transport processivity was significantly improved (Supplementary Fig. 1B, C).

Thus, the data indicate that the microtubule-targeting drug EpoD modulates the interaction of TauC3 with microtubules. They also suggest that EpoD functionally interacts with overexpressed human tau to modulate the processivity of axonal transport.

## Discussion

PTMs of tau are believed to play a critical role in the molecular events that lead to pathological changes during AD and other tauopathies. The proteolytic cleavage of tau is a special PTM because it causes an irreversible change in function that could be of potential relevance for proteins with a long half-life, such as proteins of the neuronal cytoskeleton. In this case, even minor changes in functional activity can have long-lasting chronic effects, for example due to modulating axonal transport or signal transduction properties. Several potentially pathogenic tau fragments were previously described (for a current review article, see [56]). Examples include a 35 kDa C-terminal tau fragment (Tau35) that lacks the N-terminus and is present in 4R tauopathies [57, 58] and a 17 kDa neurotoxic fragment which is generated by calpain-mediated cleavage and which lacks the microtubule-binding region [59, 60]. It must be recognized, however, that among tau fragments found in the brains of patients with tauopathic disorders, the mechanisms of neurotoxicity and their biological activities are not yet fully defined.

Here we show that (1) the proportion of tau that is proteolytically cleaved at the caspase-3 site (TauC3) doubles during aging in the hippocampus of mice. We demonstrate that (2) TauC3 induces a toxic gain of function by increasing the dwell time during the kiss-and-hop interaction with microtubules, thereby hindering axonal transport, and inducing region-specific dendritic atrophy in CA1 neurons of the hippocampus. Furthermore, we show that (3) treatment with the drug EpoD modulates the tau-microtubule-binding and improves the dynamic interaction of TauC3 with axonal microtubules.

A carboxy-terminally truncated form of tau that is generated by caspase-mediated cleavage at Asp421 has previously been observed in NFTs, neuropil threads, and dystrophic neurites in AD brain [61]. Subsequent histological staining indicated that caspase-cleavage of tau is an early event in AD pathology and occurs prior to the formation of filamentous tau in NFTs [20, 62, 63]. In addition to AD, caspase-3 activation and presence of caspase-cleaved tau was reported in Progressive Supranuclear Palsy [26], Vascular Dementia [64] and notably during normal aging as well [23]. Thus, cleavage of tau by caspase-3 or other caspases that cleave at the same position (caspase-7 and -8; [20]) may be a common driver in mediating neurodegenerative processes in a wide variety of disease- and age-related conditions. In fact, our data show that the ratio of TauC3 to total tau doubles during aging in the hippocampus of mice, a region affected early in AD, while there is no difference in the temporal cortex of the human AD brain compared to controls of similar age. We also did not observe any obvious correlation with the severity of the disease according to the Braak stages. This indicates that the generation of TauC3 during aging can promote neurodegenerative processes in diseases where age is the main risk factor, but is probably not the immediate cause of the disease.

Transgenic expression of TauC3 in the cortex and hippocampus resulted in memory deficits in mice [65] and TauC3 sensitized cultured neuronal cells to various stressful conditions [66, 67]. However, the mechanism by which caspase-cleaved tau affects neuronal physiology remained open. Tau is an abundant neuronal protein and the molar tau/tubulin ratio in neuronal cells is in a range from 0.04 to 0.06, corresponding to a protein concentration of 2.5 to 4 µM [36, 68, 69]. At physiological conditions, the vast majority of tau (>80%) is bound to microtubules [15]. Remarkably, neither elevated nor reduced tau levels affect axonal transport in vivo [70] despite tau being a potential obstacle for motor proteins [19, 71]. An explanation provides the highly dynamic interaction of tau with microtubules [15, 72, 73]. In fact, SMT revealed a rapid kiss-and-hop interaction of tau with microtubules [18] that rationalizes why tau, although binding to microtubules, does not interfere with axonal transport. This implies that conditions, which reduce the dynamicity of the tau-microtubule interaction may have a negative impact on microtubule-dependent transport. Indeed, we observed that increasing the dwell time during the kiss-and-hop interaction resulted in an impaired axonal transport of mitochondria and APP-containing vesicles. Notably, the reduced dynamicity of the interaction of TauC3 with microtubules did not affect microtubule polymerization in the axon indicating that the kinetics of tau’s microtubule interaction and its activity to promote microtubule polymerization are mechanistically distinct and can be regulated differentially.

TauC3 did not change the ratio of stationary to mobile mitochondria or vesicles and only affected the movement of the mobile fraction as it would be expected from a potential obstacle for motor proteins. Axonal transport of APP is highly processive, with longer running times in both anterograde and retrograde directions [74, 75]. Analysis of the trajectories indicated that TauC3-binding mainly reduced the processivity of vesicle transport, i.e., increased the number of stop events and changes in direction, without having a major effect on vesicle speed. This indicates that a less dynamic tau species is acting as a temporary roadblock rather than a continuous brake on vesicle transport, as shown in the scheme in Fig. 5D. Due to such a mechanism, it is conceivable that even a moderate fraction of a less dynamic tau species would suffice to negatively interfere with organelle transport. Impaired axonal transport of APP correlates with increased production of amyloid beta [76] suggesting that dysfunctional APP trafficking can directly contribute to disease pathology. Indeed, axonal transport damage is an early pathogenic event in AD and a tau-mediated impairment of axonal transport could act as a driver of Aβ-pathology.

Various experimental approaches have shown that tau expression is necessary for key neurodegenerative events to occur in AD and other pathological conditions. Examples include a study in which the reduction of endogenous tau was shown to improve amyloid beta-induced deficits in an AD mouse model [7] and the observation that tau is essential for stress-induced brain pathology [8]. Interestingly, the tau deletion also prevented stress-induced dendritic atrophy in the prefrontal cortex of mice, including tau-dependent changes in the synaptic localization of mitochondria [77] pointing to disturbed transport caused by pathologic tau species. The production of a tau species with a low dynamic microtubule interaction would establish a direct mechanistic connection between pathological conditions, a possible gain-in-toxicity of modified tau and an impairment of axonal transport. Such a mechanism also provides a mechanistic explanation of why a reduction in the amount of tau, for example through the use of tau-directed antibodies, has the potential to reduce degenerative effects.

Our data have shown that changes in the tau-microtubule interaction caused by the creation of a less dynamic tau species cause axonal transport defects and induce region-specific dendritic atrophy in CA1 neurons of the hippocampus. The modulation of the tau-microtubule interaction could therefore represent a therapeutic approach to alleviate the roadblocks caused by tau (Fig. 5D). Possible candidates are microtubule-modulating drugs such as the epothilones, which have been shown to improve cognition in a mouse model of tauopathy [78] and to increase axonal transport of mitochondria in neurons of AD model mice [79]. Indeed, we observed that treatment with low nanomolar EpoD concentrations returned the dynamic interaction of TauC3 with microtubules to levels similar to full-length tau. Notably, EpoD specifically affected the interaction of TauC3 with microtubules, but did not change the k_on_ or k_off_ rate constants of the full-length tau protein. Consequently, we also observed that the difference in vesicle transport between full-length tau and TauC3-expressing neurons was canceled.

In the presence of EpoD and both human tau constructs, however, we observed an impaired transport of APP vesicles, which is shown in a significantly reduced velocity and processivity. In the absence of exogenous tau, transport parameters improved to control levels, suggesting that EpoD and overexpressed human tau functionally interact to modulate transport properties. While EpoD normalized the interaction of TauC3 with microtubules, probably by influencing the microtubule structure, these structural changes may also override the influence of tau dynamics on transport efficiency.

The effect of EpoD on axonal transport is still controversially reported. In vitro studies have shown that several clinically used microtubule-binding drugs inhibit axonal transport [80], and a recent study showed that low nanomolar concentrations of EpoD reduce the average speed of mitochondrial transport in cortical neurons [81]. It should also be noted that an exploratory phase 1 clinical trial evaluating the tolerability and pharmacology of EpoD (BMS-241027, Bristol-Myers Squibb) in patients with mild AD ended in 2013 and evaluation of EpoD then discontinued for AD. Unfortunately, no results were provided at ClinicalsTrial.gov. Thus, the data show that microtubule-targeting drugs like EpoD are not simply stabilizers of the native structure of microtubules, but rather modulate the structure and function of axonal microtubules. However, different drugs can stabilize the microtubule lattice through different mechanisms [82]. Other modulators of tau-microtubule interaction and axonal transport have also been previously described. These include the peptide NAP and analogs, active segments of the activity-dependent neuroprotective protein that improves tau-MT interaction and axonal transport [83–86]. This offers the possibility of using certain combinations of microtubule stabilizers to regulate microtubule activity in order to optimize the therapeutic potential. Our data show that key functional aspects for such a drug are the restoration of the physiological interaction of structural MAPs with microtubules without affecting motor protein-dependent transport.

## Supplementary information

Legend Supplemental Figure 1

Supplemental Figure 1
