# Supplementary material for: Caspase-cleaved tau is senescence-associated and induces a toxic gain of function by putting a brake on axonal transport
Source: Mol Psychiatry. 2022 Apr 7;27(7):3010–23. doi: 10.1038/s41380-022-01538-2 (PMC9205779; doi:10.1038/s41380-022-01538-2)
Supplement: Supplementary file 1 — Legend Supplemental Figure 1 [file 41380_2022_1538_MOESM1_ESM.docx]

**Supplementary Figure 1. Reduction in the amount of tau prevents the impairment of axonal transport parameters by Epothilone D.**

**A.** Immunoblots of PC12 lysates transduced with mCherry-Tau or -TauC3. Lysates from 45,000 cells were loaded for each lane. For comparison, 0.1 µg of recombinant human tau protein (Tau441wt; (87)) was loaded. Molecular weight standards are indicated. Detection used Tau5 antibody; only exogenously expressed proteins were detected since endogenous tau was below the limit of detection. **B.** Immunoblots from lysates of neuronally differentiated wildtype or lentivirally transduced PC12 cells that encode shRNA to knock down endogenous rat tau gene expression (tau shRNA) showing high molecular weight rat tau isoforms (HMW Tau at ~110 kDa, Tau5), low molecular weight rat tau isoforms (LMW Tau at ~55-75 kDa) and GAPDH as loading control. Molecular mass standards are indicated. The relative amounts of HMW Tau and LMW Tau are shown on the right (mean ± SEM; n = 3). Statistically significant differences between samples determined by a t-test with Welch’s correction are indicated. ** p <0.01. **C.** Effect of EpoD on the transport of mobile APP-vesicles. The velocity and processivity of eGFP-tagged APP vesicles in wildtype PC12 cells (endogenous rat Tau) and lentivirally transduced PC12 cells to knock down endogenous rat tau gene expression (tau shRNA) are shown in the scatter plots. For comparison, transport of mobile APP-vesicles in cells overexpressing mCherry-tagged human tau (human Tau), co-expressed with eGFP-tagged APP, is plotted on the left (data from Figure 5C). Vesicle mobility was determined using an autoregressive motion algorithm. Each point represents an average value for a respective cell (mean ± SEM of n = 18 - 31 cells with 401 - 968 trajectories). Cells were treated with EpoD (5 nM) or carrier (0.01% DMSO) 1 hour before imaging. Statistically significant differences were determined by Two-Way ANOVA on log transformed data sets followed by post hoc Fischer LSD. ^#^ p <0.05; ** p <0.01; # indicates a significant difference compared to EpoD treated cells overexpressing human Tau.
