# Supplementary figures and images for: Caspase-cleaved tau is senescence-associated and induces a toxic gain of function by putting a brake on axonal transport
Source: Mol Psychiatry. 2022 Apr 7;27(7):3010–23. doi: 10.1038/s41380-022-01538-2 (PMC9205779; doi:10.1038/s41380-022-01538-2)

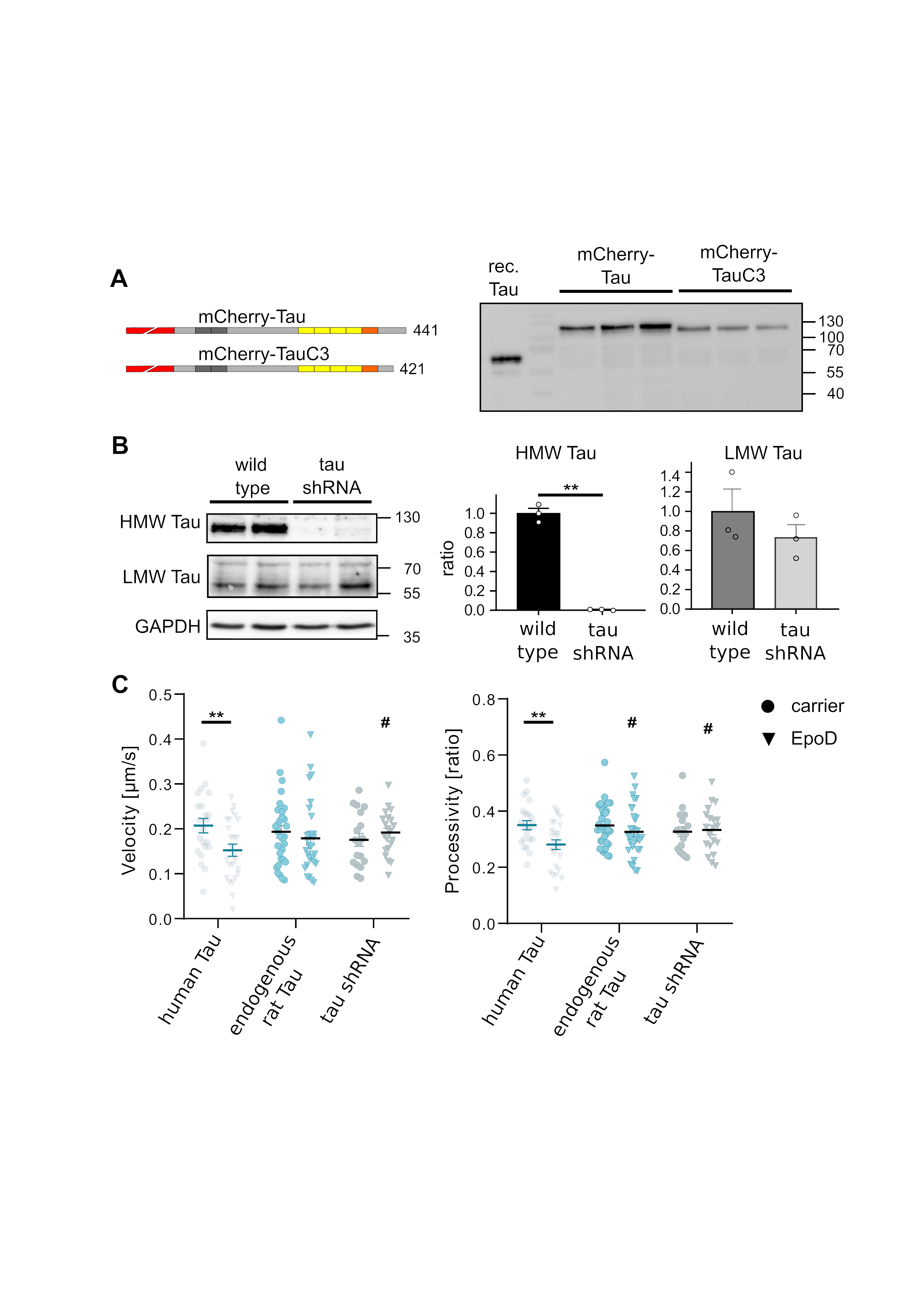

Supplement: Supplementary file 2 — Supplemental Figure 1 [file 41380_2022_1538_MOESM2_ESM.jpg]
